# Development of Novel Biocomposites with Antimicrobial-Activity-Based Magnesium-Doped Hydroxyapatite with Amoxicillin

**DOI:** 10.3390/antibiotics13100963

**Published:** 2024-10-12

**Authors:** Carmen Cimpeanu, Daniela Predoi, Carmen Steluta Ciobanu, Simona Liliana Iconaru, Krzysztof Rokosz, Mihai Valentin Predoi, Steinar Raaen, Monica Luminita Badea

**Affiliations:** 1 Faculty of Land Reclamation and Environmental Engineering, University of Agronomic Sciences and Veterinary Medicine of Bucharest, 59 Marasti Blvd., 011464 Bucharest, Romania; carmencimpeanu@yahoo.com; 2 National Institute of Materials Physics, Atomistilor Street, No. 405A, 077125 Magurele, Romania; simonaiconaru@gmail.com; 3 Faculty of Electronics and Computer Science, Koszalin University of Technology, Śniadeckich 2, PL 75-453 Koszalin, Poland; rokosz@tu.koszalin.pl; 4 Department of Mechanics, University Politehnica of Bucharest, BN 002, 313 Splaiul Independentei, Sector 6, 060042 Bucharest, Romania; predoi@gmail.com; 5 Department of Physics, Norwegian University of Science and Technology (NTNU), Realfagbygget E3-124 Høgskoleringen 5, NO 7491 Trondheim, Norway; steinar.raaen@ntnu.no; 6 Faculty of Horticulture, University of Agronomic Sciences and Veterinary Medicine, 59 Marasti Blvd., 011464 Bucharest, Romania; badea.artemisia@gmail.com

**Keywords:** magnesium, hydroxyapatite, amoxicillin, suspensions, antimicrobial activity

## Abstract

**Background/Objectives**: A biocomposite based on magnesium-doped hydroxyapatite and enriched with amoxicillin (MgHApOx) was synthesized using the coprecipitation method and is presented here for the first time. **Methods**: The stability of MgHAp and MgHApOx suspensions was evaluated by ultrasound measurements. The structure of the synthesized MgHAp and MgHApOx was examined with X-ray diffraction (XRD), Fourier transform infrared (FT-IR) spectroscopy and X-ray photoelectron spectroscopy (XPS). The crystalline structure was determined by X-ray diffraction. The FTIR data were collected in the range of 4000–400 cm^−1^. The morphology of the nanoparticles was evaluated by scanning electron microscopy (SEM). Furthermore, the biocompatible properties of MgHAp, MgHApOx and amoxicillin (Ox) suspensions were assessed using human fetal osteoblastic cells (hFOB 1.19 cell line). The antimicrobial properties of the MgHAp, MgHApOx and Ox suspension nanoparticles were assessed using the standard reference microbial strains *Staphylococcus aureus* ATCC 25923, *Escherichia coli* ATCC 25922 and *Candida albicans* ATCC 10231. **Results**: X-ray studies have shown that the biocomposite retains the characteristics of HAp and amoxicillin. The SEM assessment exhibited that the apatite contains particles at nanometric scale with acicular flakes morphology. The XRD and SEM results exhibited crystalline nanoparticles. The average crystallite size calculated from XRD analysis increased from 15.31 nm for MgHAp to 17.79 nm in the case of the MgHApOx sample. The energy-dispersive X-ray spectroscopy (EDS) and X-ray photoelectron spectroscopy (XPS) analysis highlighted the presence of the constituent elements of MgHAp and amoxicillin. Moreover, XPS confirmed the substitution of Ca^2+^ ions with Mg^2+^ and the presence of amoxicillin constituents in the MgHAp lattice. The results of the in vitro antimicrobial assay demonstrated that MgHAp, MgHApOx and Ox suspensions exhibited good antimicrobial activity against the tested microbial strains. The results showed that the antimicrobial activity of the samples was influenced by the presence of the antibiotic and also by the incubation time. **Conclusions**: The findings from the biological assays indicate that MgHAp and MgHApOx are promising candidates for the development of new biocompatible and antimicrobial agents for biomedical applications.

## 1. Introduction

Recently, significant advancements have been made in the area of biomaterials, leading to the development of new and improved multifunctional materials with excellent biological and reproducible physicochemical properties. Despite the tremendous advancements in designing and safely utilizing materials with specific properties [1,2,3,4], there are still significant limitations in the current material design strategies in numerous areas such as biomedical, environmental, engineering and more [5,6,7,8,9,10]. Over the years, research has been focused on designing novel materials whose primary function is to restore, replace or enhance the function of various organs or tissues [4,8,9,10]. One of most renowned materials considered for its promising use in the biomedical field is hydroxyapatite (HAp). Hap, having the chemical composition Ca_10_(PO_4_)_6_(OH)_2_, is one of the most promising candidates for the development of novel biocomposites. Being the primary inorganic component found in human and animal bones and teeth, HAp is known for its exceptional biological properties, including bioactivity, biocompatibility, osteoconductivity and non-toxicity [11,12,13,14]. Over the years, composite materials based on hydroxyapatite have generated great research interest due to their exquisite biological features. According to previously reported studies, the use of various elements as dopants has been shown to enhance both the physicochemical and biological properties of hydroxyapatite nanoparticles, making this material an ideal candidate for the development of novel innovative biocomposites suitable for a large range of biomedical applications [15,16,17,18]. Among the ions proposed as dopants for HAp, magnesium (Mg^2+^) stands out due to its exceptional biological properties. Magnesium is a highly abundant cation in the human body and plays a crucial role in biological processes such as protein synthesis and glycolysis. Also, it is known that it can activate enzyme systems and regulate the crystallization of biological calcium phosphates [18,19]. In their studies, Zhao et al. [20] demonstrated that coating a surface with Mg/HAp had the ability to enhance the osteogenic differentiation of pre-osteoblasts and also helped promote the early bone osseointegration process. More than that, earlier reported studies highlighted that hydroxyapatite composite materials exhibit non-cytotoxic effects when tested on different types of cells such as human colon cancer (HCT-8) and primary osteoblast (hFOB 1.19) cell lines [16]. The incorporation of Mg^2+^ into pulp-capping materials has been suggested as a promising strategy for developing materials that promote dental tissue regeneration by Li et al. [21] based on their findings. Additionally, magnesium in hydroxyapatite-based compounds has been reported to enhance the in vitro antimicrobial activity of HAp [22]. On the other hand, antibiotic resistance has emerged as one of the most urgent global health threats, as highlighted by the World Economic Forum. It has been reported that antibiotic-resistant bacteria are responsible for a large number of deaths and excessive healthcare costs annually. In this context, the need for novel antimicrobial agents is one of the principal targets of researcher communities. Amoxicillin is a widely used antibiotic for treating various bacterial infections. It has been reported to be particularly effective against respiratory tract infections, ear infections, sinusitis, and urinary tract infections. It has the ability to inhibit the growth of various bacterial strains, thus helping to alleviate symptoms such as pain, fever, and inflammation. Its broad-spectrum action makes it a valuable choice for both children and adults in combating common infections [23,24,25]. In this context, the development of a new composite enriched with amoxicillin could represent a valuable candidate as an antimicrobial agent. Thus, the novelty of this paper mainly consists of the development for the first time of magnesium-doped hydroxyapatite composites enriched with amoxicillin (MgHApOx) by an adapted method. Moreover, we report for the first time the results of the novel MgHApOx complex characterization. The development of magnesium-doped hydroxyapatite composites enriched with amoxicillin represents a significant innovation in the field of biomedical materials. These novel materials manage to bring together the enhanced structural properties of magnesium-doped hydroxyapatite with the significant antimicrobial efficacy of amoxicillin. Incorporating magnesium into hydroxyapatite, which is known as a biocompatible and osteoconductive ceramic, can improve its properties and promote better cell adhesion and proliferation, making it highly suitable for bone tissue engineering applications. Meanwhile, the addition of amoxicillin, a well-known broad-spectrum antibiotic, helps ensure the material’s ability to stop the development of potential bacterial infections, which could help reduce the risk of post-operatory complications. This dual-functionality approach provides a promising strategy for the future development of advanced bone graft materials that support both rapid osseointegration and localized infection control and will address the current critical challenges in orthopedic and dental procedures.

The aim of this study was to obtain for the first time the magnesium-doped hydroxyapatite composites enriched with amoxicillin (MgHApOx) by the coprecipitation adapted method. In order to give nanohydroxyapatite (HAp) more biological functions and a greater scope of applicability in the biomedical field, this research studied magnesium-doped hydroxyapatite enriched with amoxicillin (MgHApOx) in a suspension for the first time. In this study, the stability, structure, morphology and chemical composition of MgHAp and MgHApOx were investigated by ultrasonic measurements, X-ray diffraction (XRD), scanning electron microscopy (SEM) and energy-dispersive X-ray spectroscopy (EDS), Fourier transform infrared spectroscopy (FTIR) and X-ray photoelectron spectroscopy (XPS). The results showed that the enrichment of MgHAp samples with amoxicillin leads to slight changes in the stability, average crystallite size and morphology.

## 2. Results and Discussions

**Figure 1:**
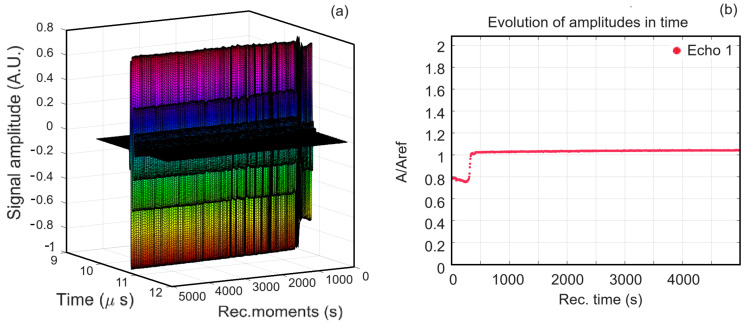
Time evolution of the recorded signals of MgHAp suspension from left to right over 5000 s (**a**); Recorded signals amplitudes during the experiment for MgHAp suspension (**b**).

**Figure 2:**
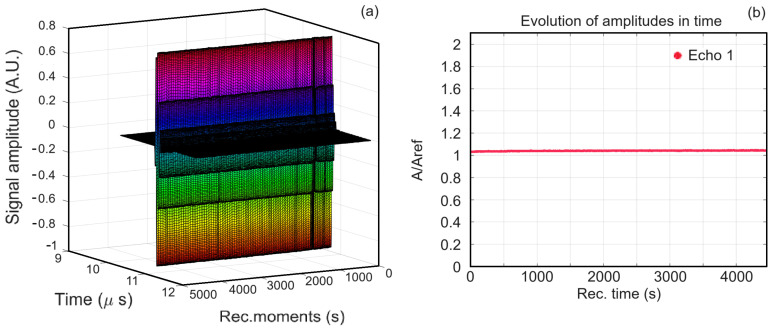
Time evolution of the recorded signals of MgHApOx suspension from left to right over 4500 s (**a**); Recorded signals amplitudes during the experiment for MgHApOx suspension (**b**).

Figure 1a and Figure 2a show a superposition of the signals recorded in the case of MgHAp suspensions (1000 signals) and MgHApOx (900 signals), respectively. In both figures, from right to left, they are plotted as water flow, with all these signals covering 5000 (MgHAp) and 4500 (MgHApOx) seconds of process evolution. The rapid initial evolution of the amplitudes in the case of the MgHAp sample is followed by an almost constant amplitudes phase being detailed in Figure 1b. It can be seen that, initially, we have a small decrease in the initial amplitude of the signal, which is followed by a sudden increase to a constant amplitude. In the case of the MgHApOx sample, we observe that the amplitudes of the recorded signals are almost constant during the experiment (Figure 2b). This ultrasonic signal amplitude indicates the global evolution in the time of the suspension, pertaining to its speed of sedimentation and related to stability. The relative amplitude depends on the compressibility coefficient of the diphasic medium compared to that of distilled water. An amplitude slightly higher than one indicates a higher average compressibility coefficient than in pure water.

**Figure 3:**
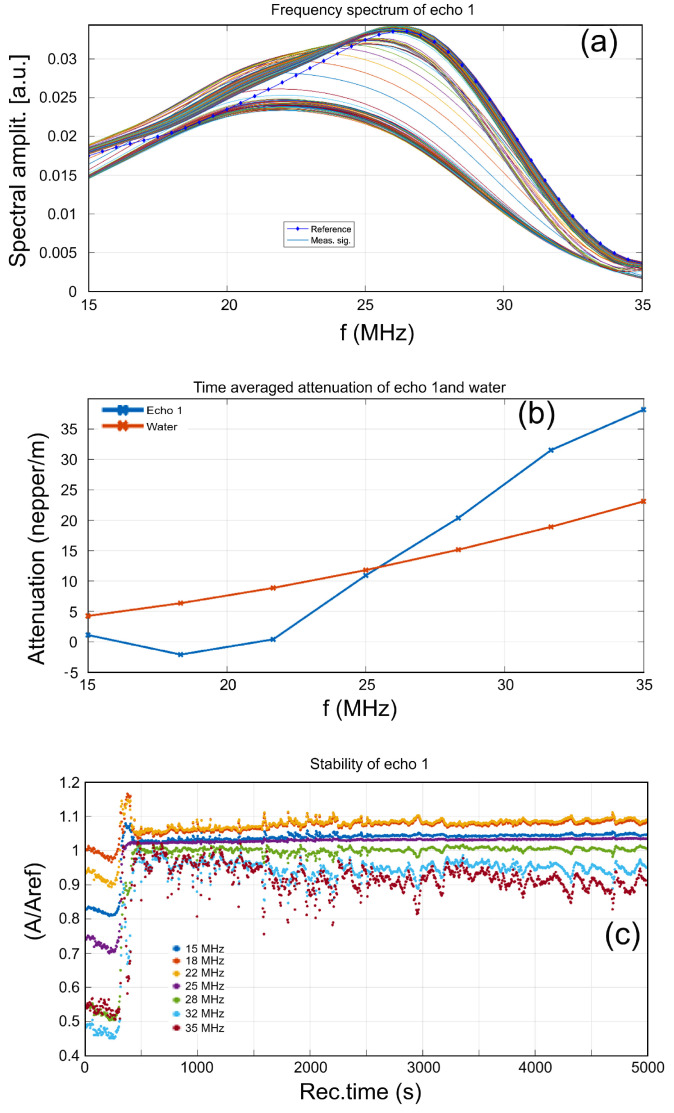
Spectral amplitudes of all recorded signals (**a**), time-averaged attenuation for the investigated frequency range (**b**) and relative spectral amplitudes vs. time (**c**) of MgHAp sample.

**Figure 4:**
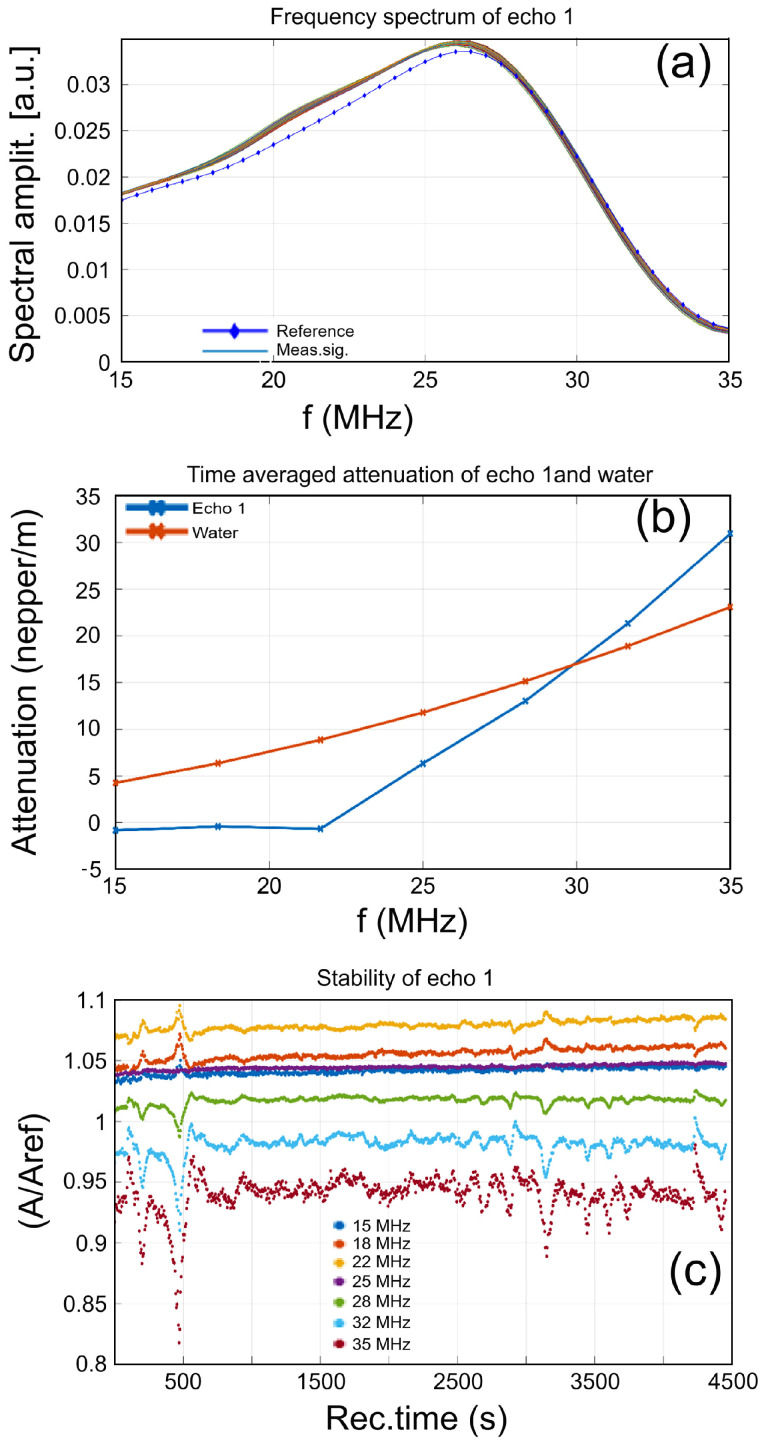
Spectral amplitudes of all recorded signals (**a**), time-averaged attenuation for the investigated frequency range (**b**) and relative spectral amplitudes vs. time (**c**) of MgHApOx sample.

The temporal change of properties manifests in the variation of the frequency spectra of all recorded MgHAp and MgHApOx sample signals. These spectra are shown in Figure 3a and Figure 4a. For comparison, the spectrum of the reference liquid (double-distilled water, dotted blue line) was also plotted. The temporal evolution of signal frequency spectra of the MgHAp sample (Figure 3a), which is related to the properties of the suspension in front of the transducers, is represented by two zones of concentration of the 1000 spectral curves. The lower set of spectra corresponds to the initial phase of decreasing amplitudes and shows important differences compared to the spectra of the reference liquid: lower amplitudes in general, only in the 18–20 MHz range, the spectra coincide with that of the reference liquid. The peak for these spectra is at lower frequencies (~22 MHz). Then, during the rapid evolution phase, a few spectra represent the evolution towards the stable state, in which many spectrums get closer to the spectrum of the reference liquid, which has a peak at 26.2 MHz. However, the remaining particles in suspension lead to higher amplitudes compared to the reference liquid, visible in the lower frequencies domain (15–25 MHz). The existence of these two patterns indicates a major modification of the suspension structure, due to the important sedimentation process, from the spectra of larger particles sedimentation to the spectra of the stable suspension of smaller particles.

On the other hand, the signal frequency spectra of the MgHApOx sample (Figure 4a) remain very close together during the experiment. The peak for these spectra is at lower frequencies (~26 MHz) compared to the spectrum of the reference liquid, which has a peak at 26.2 MHz. For frequencies below the peak, the amplitudes of the suspension remain stable above the values for the reference liquid, whereas those corresponding to higher frequencies are practically superposed on the spectrum of the reference liquid. This remark indicates the presence of highly diluted particles with a relatively larger dimension but very stable properties during the experiment, lacking any detectable sedimentation process.

The ultrasonic signals are attenuated by the suspension. The time-averaged attenuation plot is shown in Figure 3b and Figure 4b. Compared to the standard attenuation in the reference liquid (red dotted line), the attenuation is larger for the MgHAp sample (Figure 3b) in the higher frequency ranges, reaching 38 nepper/m at 35 MHz. In the frequency range of 15–25 MHz, the attenuation is lower than the attenuation in the reference liquid. Moreover, compared against the standard attenuation in the reference liquid (red dotted line), the attenuation is larger for the MgHApOx sample (Figure 4b) in the higher frequency ranges, reaching 31 nepper/m at 35 MHz. In the frequency range of 15–30 MHz, the attenuation is lower than the attenuation in the reference liquid.

Another MgHAp and MgHApOx suspension characteristic is spectral stability, representing the amplitude of the frequency component of each spectrum, as a function of time (Figure 3c and Figure 4c). Some remarkable features can be associated with these MgHAp and MgHApOx samples.

In Figure 3c (MgHAp sample), during the first 250 s, all spectral amplitudes decrease slowly, a fact that can be attributed to the settlement of the particles in suspension. Then, at 250 s, the amplitudes rise sharply due to the passage of the sedimentation-free surface in front of the transducers. The suspension remains unstable during the entire experiment. Only the frequency component of 25 MHz shows a stable and slowly increasing amplitude. For all the other frequencies in the spectrums, there are rapid random variations. This indicates that the particles in suspension tend to aggregate and pass in front of the transducers during the entire experiment. In Figure 4c (MgHApOx sample), we can observe that during the first 500 s, the spectral amplitudes of the higher frequencies (28–35 MHz) have two small dips, a fact that can be attributed to the nonuniform settlement of the particles in suspension. Then, these amplitudes continue to exhibit oscillations all along the experiment, being explained by the formation of clusters of the smallest particles in suspension, which are sensitive to higher-frequency components in the ultrasonic signal. On the contrary, the central frequency (25 MHz) of the spectrum keeps a very uniform value of the relative amplitude (1.04) throughout the experiment.

On the contrary, for the lower frequencies (15–22 MHz) of the spectrum, the amplitudes increase with increasing frequency, but they all have peaks before t = 500 s, followed by small oscillations throughout the experiment. This relative balance of spectral amplitudes, with some rations above 1 and others below, explains the overall stable behavior of the suspension. The MgHAp sample becomes relatively stable after the rapid evolution lasting 500 s, as proved by the stability s = 0.00032 s^−1^. A is the signal amplitude, with a bar above indicating averaging. The short-lasting variations of amplitudes are averaged, and this fact explains the obtained good stability parameter. The MgHApOx sample is extremely stable overall, as proved by the stability parameter s = 6.85 × 10^−6^ s^−1^. A stability parameter of zero indicates a perfectly stable liquid medium, with no time evolution of the signal amplitude, whereas S = 0.001 corresponds to a rather unstable dispersion, with a variation of 0.1% of the averaged signal amplitude in 1 s.

Stability information for the MgHAp and MgHApOx suspensions was obtained through ultrasound measurement (US) and zeta potential (ZP) studies. Ultrasonic measurements on the concentrated suspensions indicated relative stability (s = 0.00032 s^−1^) for the MgHAp sample, while the MgHApOx suspension was extremely stable (s = 6.85 × 10^−6^ s^−1^). In order to estimate the zeta potential, the MgHAp and MgHApOx concentrated suspensions were diluted 20 times. The zeta potential measured for the suspension of MgHAp was −14.8 mV, while the potential measured for MgHApOx suspension was −42.6 mV. The low ζ-potential value for the MgHAp sample indicates that the suspension is unstable. In accordance with precedent studies [26,27,28,29], colloids with low ζ-potential are prone to flocculation, suggesting early signs of instability. The zeta potential, which indicates the total charge on the nanoparticle surface, is considered optimal for achieving physical colloidal stability when it is around ±30 mV [30,31,32]. The results obtained regarding the behavior of the studied samples are in agreement with previous research [26,27,28,29,33,34]. Previous studies showed that the zeta potential for suspensions of magnesium-doped hydroxyapatite nanoparticles in dextran matrix was −32.3 mV [33], while for the suspension of magnesium-doped hydroxyapatite (x_Mg_ = 0.1) nanoparticles, the zeta potential was −17.77 ± 3.4 mV [34]. The difference between the zeta potential obtained for the 10MgHAp [34] and 5MgHAp suspensions (which are the object of the present study) could be due to the magnesium concentration. The stability of MgHAp suspensions increases with the concentration of magnesium. In line with previous studies by Suganthi et al. [35], which indicated that a suspension is stable if the absolute zeta potential exceeds 30 mV, it can be concluded that the MgHApOx suspension exhibits very good stability.

**Figure 5:**
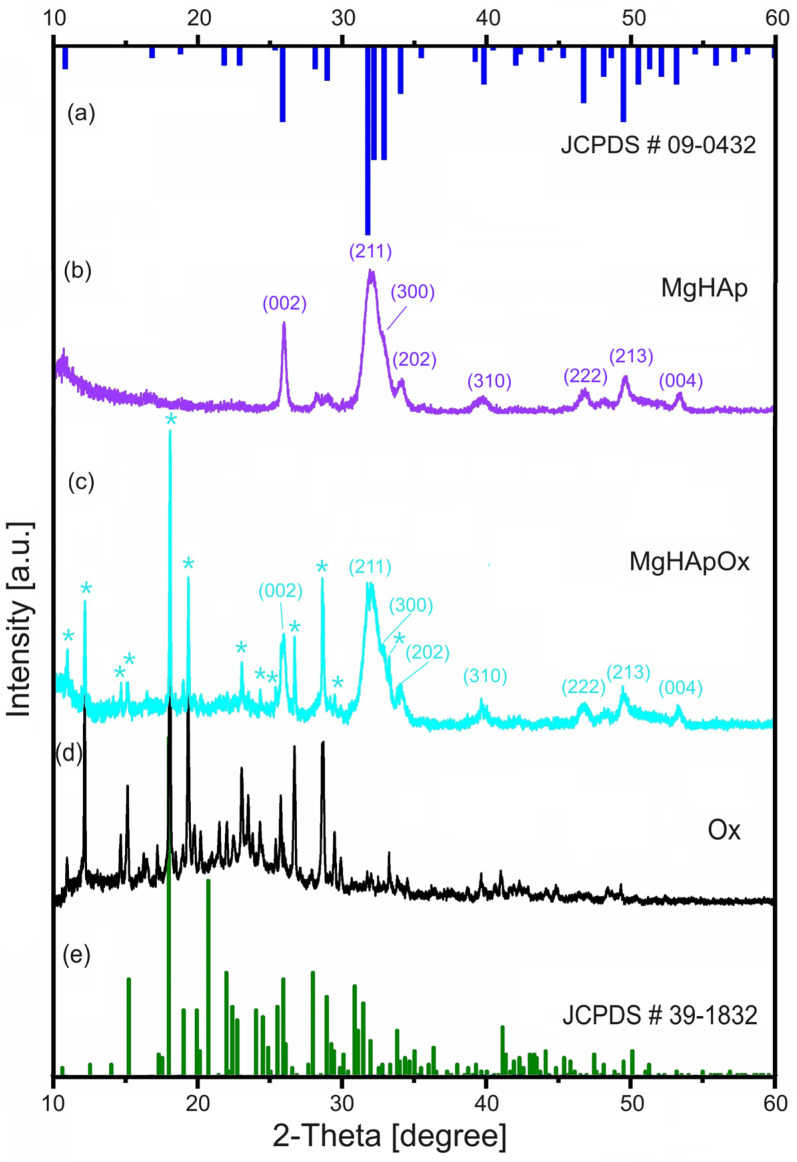
XRD patterns of MgHAp (**b**), MgHApOx (**c**) and Ox (**d**) samples. The JCPDS # 09-0432 of HAp (**a**) and JCPDS # 39-1832 of Ox (**e**). The * indicates the maxima associated with amoxicillin structure.

**Figure 6:**
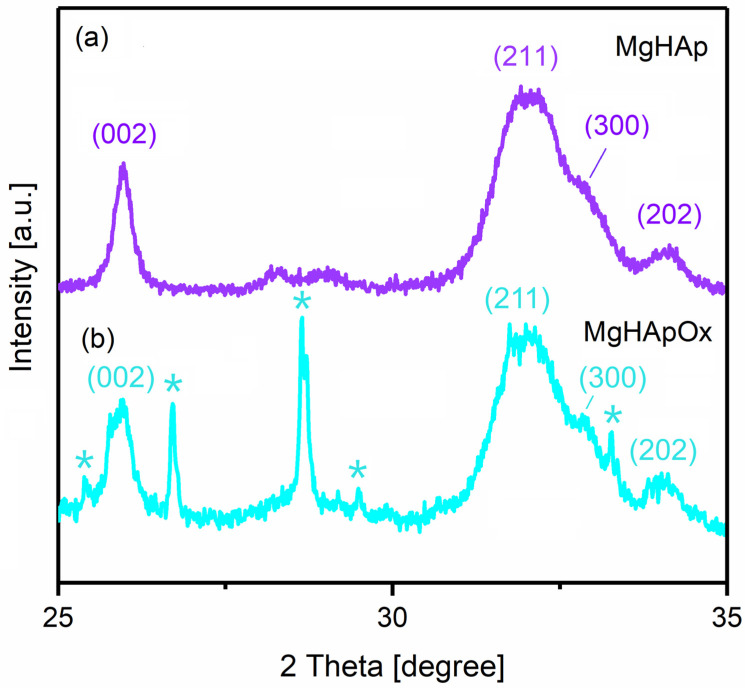
XRD patterns of MgHAp (**a**), MgHApOx (**b**) relative shift on 2θ range of 25–35°. The * indicates the maxima associated with amoxicillin structure.

The XRD patterns of the MgHAp, MgHApOx and Ox samples are shown in Figure 5. The XRD patterns of the MgHAp sample (Figure 5b) showed reflections due to the planes that correspond to the hydroxyapatite with a hexagonal lattice with a P63/m space group (JCPDF # 09-0432). On the other hand, the XRD patterns of the MgHApOx sample (Figure 5c) revealed the reflections due to the planes that correspond to the hydroxyapatite (JCPDF # 09-0432) and amoxicillin (JCPDS # 39-1832). The peaks assigned to the (0 0 2), (2 1 1), (1 1 2), (3 0 0), (2 0 2), (3 1 0) and (0 0 4) reflection plans were observed in the both XRD patterns of the MgHAp and MgHApOx. Intense diffraction peaks corresponding to amoxicillin were also observed in the MgHApOx sample. Moreover, the changes in the crystal structure show the substitution effect of amoxicillin in the MgHApOx sample. The diffraction peaks of MgHApOx shifted to a smaller angle than those of MgHAp (Figure 6).

In agreement with previous studies [36], this behavior suggests a significant incorporation of amoxicillin into the MgHAp lattice and an increment in the unit cell of the HAp in the a- and c-axes as well as the unit cell volume. Table 1 presents the average crystallite size calculated using the Scherer equation, d-spacing, lattice parameters and unit cell volume of both analyzed samples.

It was observed that the average crystallite size increased in the case of the MgHApOx sample. This behavior could be due to the weakening of the hydrogen bonds that lead to the flexibility of amoxicillin and implicitly to a slight increase in the average size of the crystallite. In agreement with previously published results [37], the crystallite size could also be influenced by the intermolecular hydrogen bonds (in our case, the hydrogen bonds from amoxicillin). This behavior is also supported by a slight increase in the distance d, the parameters of the unit cell and the volume of the cell.

**Figure 7:**
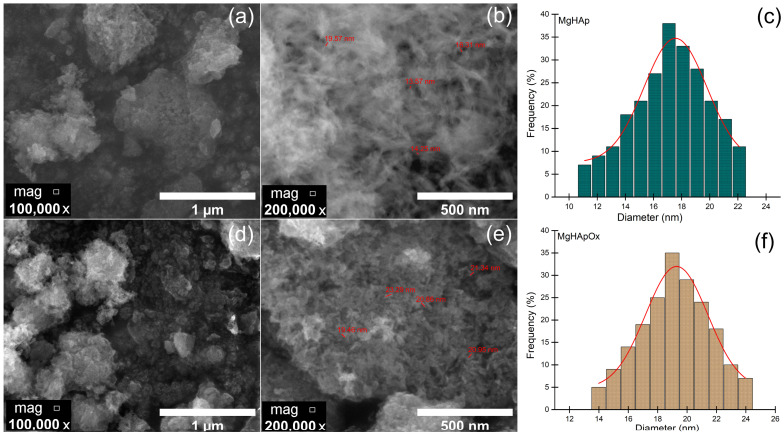
(**a**,**b**) SEM micrographs obtained at ×100,000 and at ×200,000 for MgHAp sample; (**d**,**e**) SEM micrographs obtained at ×100,000 and at ×200,000 for MgHApOx sample. (**c**,**f**) particle size distribution obtained for MgHAp and MgHApOx.

Figure 7 depicts both the SEM micrographs and the mean particle diameter distribution obtained for the MgHAp and MgHApOx samples. For both samples, the SEM micrographs were obtained at two different magnifications: ×100,000 (Figure 7a,d) and ×200,000 (Figure 7b,e). Firstly, our results underline that the MgHAp samples consist of nanoparticles with acicular flake morphology. As can be seen in the SEM micrographs of MgHApOx, the presence of amoxicillin induces some changes in the nanoparticles’ morphology. Therefore, the MgHApOx exhibits a well-defined ellipsoidal morphology.

Figure 7c,f reveal the mean particle diameter distribution (from SEM) obtained for the MgHAp and MgHApOx samples. For the MgHAp sample, a mean particle diameter of 17.55 ± 2 nm was obtained. Meanwhile, for the MgHApOx sample, a mean particle diameter of 19.28 ± 2 nm was obtained. Thus, the nanometric dimensions of the MgHAp and MgHApOx samples are sustained and produce SEM results that are in good concordance with the XRD results.

**Figure 8:**
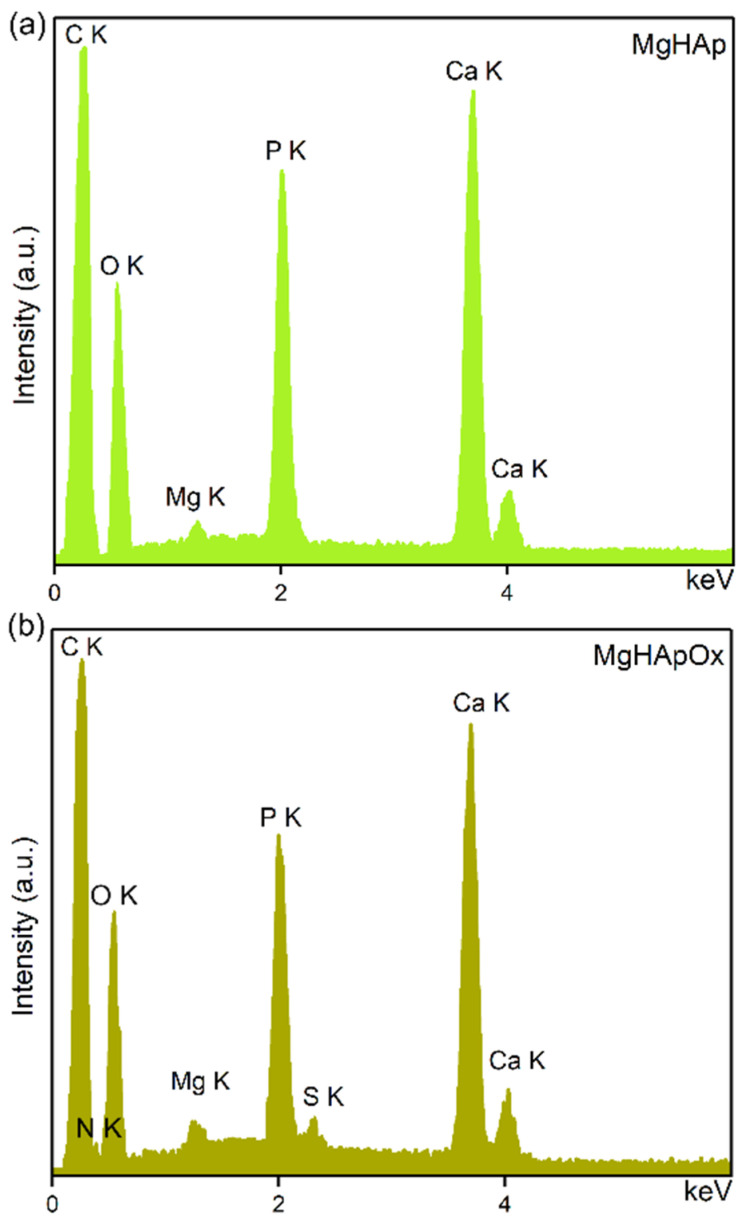
Energy-dispersive X-ray spectroscopy spectra of (**a**) MgHAp and (**b**) MgHApOx.

Data about the chemical composition of the studied specimens (MgHAp and MgHApOx) were obtained by EDS studies (Figure 8). In the energy-dispersive X-ray spectroscopy spectra of the MgHAp sample, we observe the presence of lines that are characteristic of calcium (Ca), magnesium (Mg), oxygen (O) and phosphorus (P). These are typical chemical elements characteristic of the MgHAp structure. The energy-dispersive X-ray spectroscopy spectra of the MgHApOx sample confirm the presence of MgHAp and Ox in the sample. In the MgHApOx-EDS spectra, we observe lines that belong to both compounds (Mg, P, O, C, Ca, S and N). In the energy-dispersive X-ray spectroscopy spectra of the MgHApOx sample, we observe lines that appear due to the Ox presence in the sample (sulfur (S) and nitrogen (N)). The obtained EDS spectra testify to the good purity of the MgHAp and MgHApOx samples. The results of the energy-dispersive X-ray spectroscopy studies are in good agreement with the ones obtained by X-ray photoelectron spectroscopy studies (XPS).

**Figure 9:**
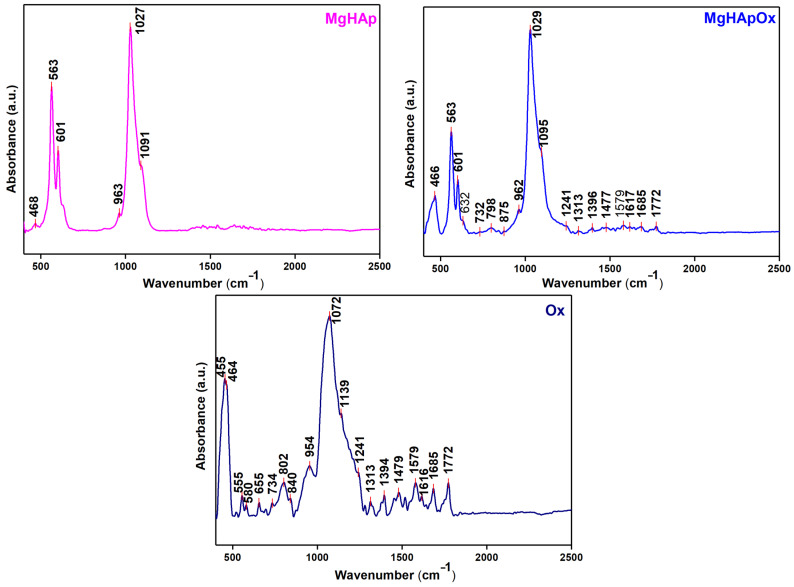
FTIR general spectra of MgHAp, MgHApOx and Ox.

The FTIR spectra obtained for MgHAp, MgHApOx and Ox are revealed in Figure 9. The FTIR spectra of the MgHAp and MgHApOx are dominated mainly by the peaks that are characteristic of the functional groups from HAp. Thus, the peak observed at ~963 cm^−1^ could be attributed to the ν_1_ vibration of phosphate groups from HAp [38]. The peaks noticed at ~468 cm^−1^ belong to the ν_2_ of phosphate groups. On the other hand, the peaks found at ~563 cm^−1^, ~601 cm^−1^, ~1027 cm^−1^ and around 1091 cm^−1^ are specific to the ν_4_ and ν_3_ vibrations of the phosphate group from HAp [38]. Furthermore, the shoulder observed at ~876 cm^−1^ could appear due to the carbonate group in the MgHAp sample [38]. In the case of the MgHApOx, we noticed that the HAp peak positions were slightly shifted, probably due to the presence of Ox. Moreover, among the specific peaks of the HAp in the FTIR the MgHApOx are noticed and the specific peaks of Ox. These results indicate that amoxicillin interacts effectively with HAp.

In the FTIR spectra of amoxicillin (Ox) revealed in Figure 9, we noticed maxima that are specific to their structure (amoxicillin). The peak observed at ~1394 cm^−1^ belongs to the NH bend CB stretch combination band and NH^3+^ symmetric deformation of Ox [39]. We also noticed other peaks that are characteristic of Ox at ~1685 cm^−1^ (attributed to νC=O (amide)), ~1616 cm^−1^ (arise due to C=C (aromatic)) and ~1772 cm^−1^ (belongs to νC=O (β-lactamic ring)) [40,41,42,43,44]. Also, the peak noticed at ~1579 cm^−1^ is characteristic of (νasCOO^−^) [41]. The peak observed at ~1072 cm^−1^ could be attributed to C–N stretching vibrations from Ox [42,43,44]. Furthermore, at ~1394 cm^−1^ and ~1479 cm^−1^ appear peaks that could arise due to the -CH_2_ bending and N–H bending vibrations of Ox [42]. The presence of the absorption peaks between the 1800 and 1650 cm^−1^ spectral region of the MgHApOx FTIR spectra clearly indicate the presence of carbonyl (-C=O) groups, corresponding to both the beta-lactam ring and the amide functional group from amoxicillin.

**Figure 10:**
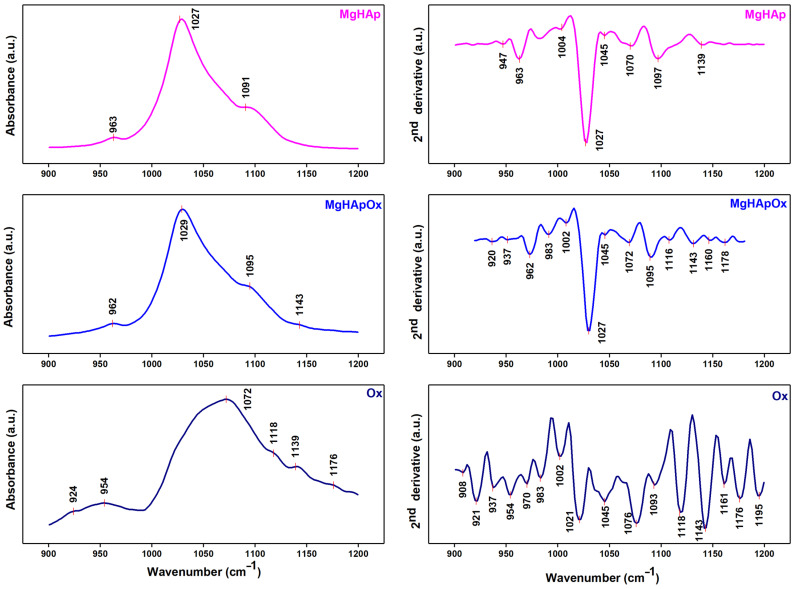
FTIR spectra of MgHAp, MgHApOx and Ox obtained between 900 and 1200 cm^−1^ and their second derivative curve.

Figure 10 reveals the second derivative spectra of MgHAp, MgHApOx and Ox samples obtained for the 900–1200 cm^−1^ spectral domain. Usually, in the case of the MgHAp sample, the vibration bands (ν_1_ and ν_3_) that belong to the phosphate groups that form the HAp structure could be found in this spectral domain. Therefore, in the FTIR, the second derivative spectra of the MgHAp are dominated by the intense band observed at ~1027 cm^−1^, which is specific to the ν_3_ vibration of the phosphate group. Also, other maxima at ~1004 cm^−1^, ~1045 cm^−1^, ~1070 cm^−1^ and ~1097 cm^−1^ that could be attributed to the ν_3_ vibration of the phosphate group from Hap could be observed in the same spectra. Another important peak that belongs to ν_1_ vibration of the HAp (phosphate group) was found at ~ 963 cm^−1^. The FTIR second derivative spectra of Ox were obtained in order to highlight that the additional peaks observed in the FTIR second derivative spectra of MgHApOx appear due to the Ox presence in the samples. Therefore, in the Ox spectra presented in Figure 10, we observed the presence of various peaks that belong to the vibration of functional groups from Ox. Multiple peaks could be observed between the 1200 and 900 cm^−1^ regions of the Ox FTIR second derivative spectra that correspond to the characteristic in-plane C–H bending vibrations of aromatic compounds [43,44]. In the case of the MgHApOx, it could be noticed that the peaks that are associated with the HAp and Ox vibration are slightly shifted compared with their position in the FTIR second derivative reference spectra (MgHAp and Ox, respectively).

Thus, the peaks observed in the second derivative spectra of MgHApOx (Figure 10) are specific to both functional groups from OX and HAp. In this case, the peaks from ~1027 cm^−1^, ~1002 cm^−1^, ~1045 cm^−1^, ~1072 cm^−1^ and ~1095 cm^−1^ are characteristic to the ν_3_ vibration of the phosphate group. Meanwhile, the ν_1_ vibration peak of the phosphate group could be observed at ~962 cm^−1^ in the second derivative spectra of MgHApOx.

**Figure 11:**
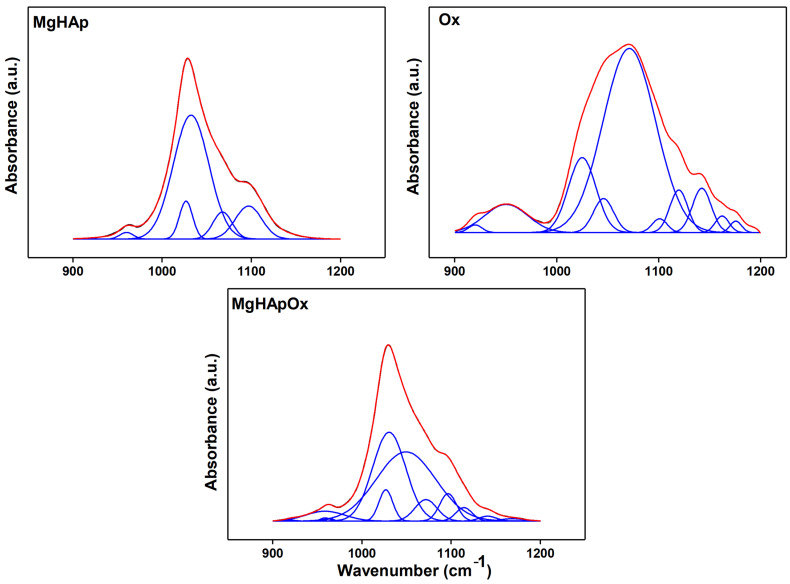
Deconvoluted FTIR spectra of the MgHAp, Ox and MgHApOx obtained in the 900–1200 cm^−1^ spectral domain.

Figure 11 presents the FTIR deconvoluted spectra of MgHAp, MgHApOx and Ox samples obtained for the 900–1200 cm^−1^ spectral region. Figure 11 presents the overlay of experimental and calculated data (red line), along with the individual subbands (blue lines) obtained using curve fitting analysis of the MgHAp, MgHApOx and Ox samples between the 900 and 1200 cm^−1^ regions. To obtain a satisfactory fit, we needed 5 subbands for the MgHAp sample, 13 subbands for the MgHApOx sample and 13 subbands for the Ox sample. The maximums of the five subbands used for the fit of MgHAp are observed at 960 cm^−1^, 1026 cm^−1^, 1032 cm^−1^, 1067 cm^−1^ and 1098 cm^−1^. Moreover, it can be observed that the main vibrational bands that are found in the MgHApOx spectra in the studied spectral domain are attributed to hydroxyapatite (specifically phosphate groups) and to the Ox structure. Thus, the FTIR studies confirm the presence of both HAp and Ox in the MgHApOx powders, indicating their effective interaction.

**Figure 12:**
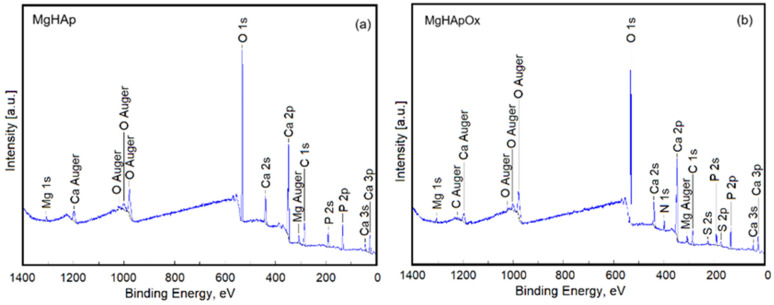
General XPS spectra of MgHAp (**a**) and MgHApOx (**b**) samples.

XPS investigations of MgHAp (Figure 12a) and MgHApOx (Figure 12b) were conducted to confirm the presence of mg and amoxicillin in the HAp lattice. The general XPS spectra of MgHAp and MgHApOx presented in Figure 12a,b highlight the presence of the constituent elements of MgHAp (O, Ca, P and Mg) in both analyzed samples. Also, the constituent elements of amoxicillin (C, N and S) were observed in the general XPS spectrum of MgHApOx, which is presented in Figure 12b. For both analyzed samples, the energy reference was established as the C–C line located at approximately 284.8 eV.

**Figure 13:**
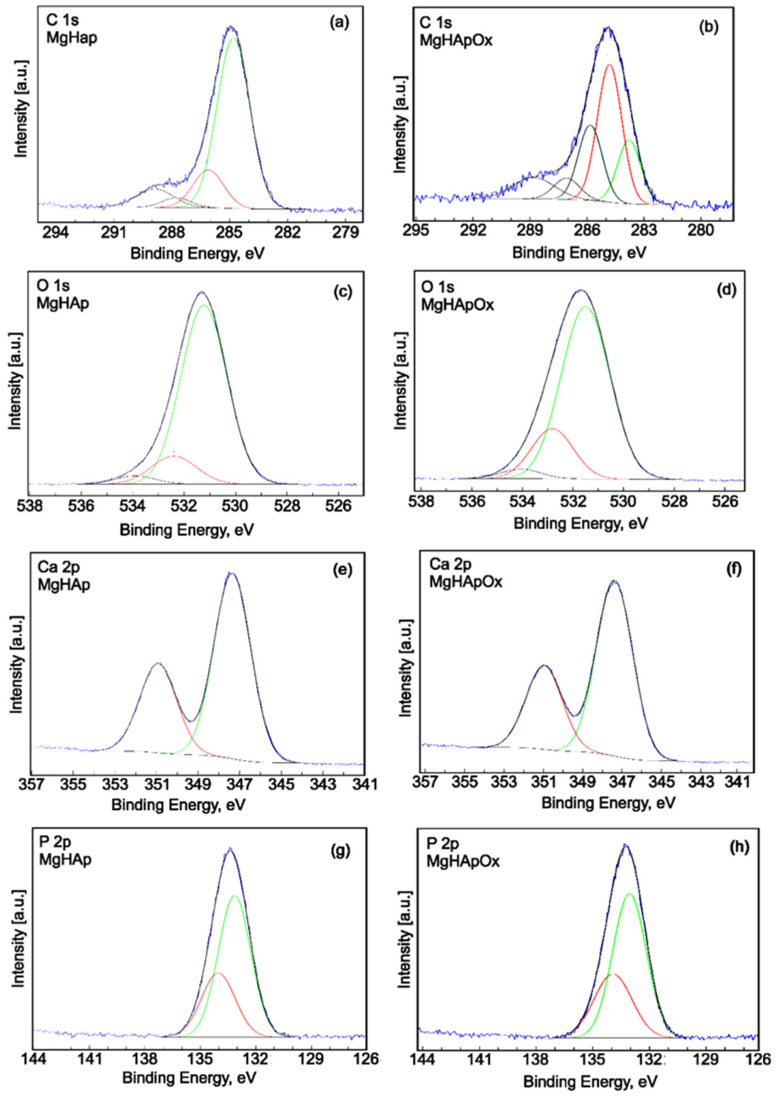
High-resolution XPS spectra and curve-fitting results of C 1s for MgHAp (**a**) and MgHApOx (**b**); O1s for MgHAp (**c**) and MgHApOx (**d**); Ca2p for MgHAp (**e**) and MgHApOx (**f**); P2p for MgHAp (**g**) and MgHApOx (**h**).

The high-resolution XPS spectra of C1s, O 1s, Ca 2p and P 2p for MgHAp and MgHApOX samples were presented in Figure 13a–h. The high-resolution XPS spectrum of C1s for the MgHAp sample (Figure 13a) shows four distinct signals. The four distinct signals in the XPS spectrum of C1s of the MgHAp sample were identified at the binding energy (BE) of 284.80, 286.10, 287.75 and 288.87 eV, respectively. These bond energies were associated with the single bonds C–C (284.80 eV), C–O (286.10 eV), the double bonds C=C (287.75 eV) and O–C–O (287.75 eV) and –COOR groups (288.87 eV) representing contaminants. The XPS high-resolution spectrum of C1s for the MgHApOx sample (Figure 13b) showed five distinct signals. The five distinct signals in the XPS spectrum of the C1s of the MgHApOx sample were identified at the binding energy (BE) of 283.79, 284.81, 285.64, 287.24 and 288.29 eV, respectively. The signal observed at a BE of around 283.79 eV was allocated to C–C and C–S single bonds. The signal observed at a BE of 284.81 eV was assigned to C=C bonds (sp2 hybridization). The signal identified at 285.64 eV was allocated to C–N and C–O single bonds, while the signal observed at 287.24 eV was assigned to C=O and O–C–O double bonds. The signal observed at 288.29 eV was assigned to –COOR groups representing contaminants.

The high-resolution XPS spectra of O 1s for MgHAp (Figure 13c) and MgHApOx (Figure 13d) samples were presented. For both samples, the high-resolution XPS spectra of O 1s revealed three distinct signals. The first signal revealed at BE of 531.23 eV (MgHAp) and 531.50 eV (MgHApOx) was associated with a Ca–O bond [45] of hydroxyapatite (HAp) and also includes C=O double bonds. On the other hand, previous studies [46,47,48] have shown that the binding energy of chemisorbed oxygen species (O–) is in the range of 531.0–531.5 eV. The second signal revealed at BEs of 532.4 eV (MgHAp) and 532.6 eV (MgHApOx) could be assigned to the P–O bonds [45] and simple C–O bonds. The third signal observed in the XPS spectra of O 1s was identified at binding energies of 533.90 eV (MgHAp) and 534.01 eV (MgHApOx) and can be allocated to single O–H bonds [49,50].

The high-resolution XPS spectrum of Ca2p for MgHAp (Figure 13e) and MgHApOx (Figure 13f) showed two distinct signals. The peaks of Ca 2p3/2 and Ca 2p1/2 for the MgHAp sample were observed at 347.35 and 350.91 eV, respectively, assigned to the tetravalent state (Ca^2+^). For the MgHApOx sample, the BE of the Ca 2p3/2 and Ca 2p1/2 states increased slightly to 347.38 and 350.96 eV, respectively. This behavior could be attributed to the presence of amoxicillin (N and S ions) in the MgHAp lattice. For both samples, the Ca2p doublet with the two specific lines (2p3/2 and 2p1/2) are separated by approximately 3.6 eV, with the close area ratio being 2:1. The binding energy is specific to hydroxyapatite in both samples [51].

The high-resolution XPS spectrum of P 2p for MgHAp and MgHApOx are presented in Figure 13g and Figure 13h, respectively. The peaks of P 2p3/2 and P 2p1/2 for the MgHAp sample were observed at 133.15 and 134.03 eV. For the MgHApOx sample, the peaks of P 2p3/2 and P 2p1/2 were observed at 133.05 and 133.93 eV. The P2p doublet with two specific lines (2p3/2 and 2p1/2) spaced at approximately 0.9 eV and with an area ratio close to 2:1 was observed in the two analyzed samples (MgHAp and MgHApOx). The binding energy of the doublet is specific to hydroxyapatite [52,53].

**Figure 14:**
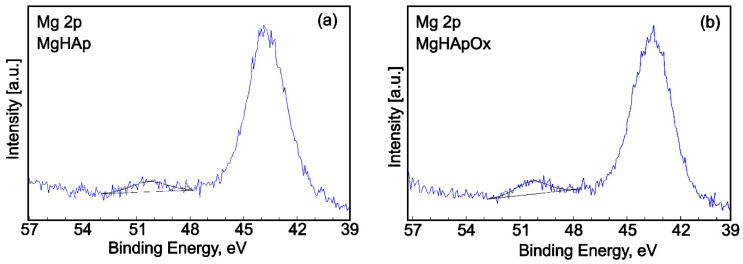
High-resolution XPS spectra and curve-fitting results of Mg 2p for MgHAp (**a**) and MgHApOx (**b**).

The high-resolution XPS spectra of Mg 2p showed one distinct signal for both MgHAp (Figure 14a) and MgHApOx (Figure 14b) samples. Consistent with the findings of S. Ardizzone et al. [54], the binding energy (BE) of Mg 2p at 50.36 eV (MgHAp sample) and 50.34 eV (MgHApOx sample) could indicate the presence of Mg^2+^ bonds with the PO_4_^2−^ group. On the other hand, metallic magnesium with a BE of around 49 eV [55] was not detected.

**Figure 15:**
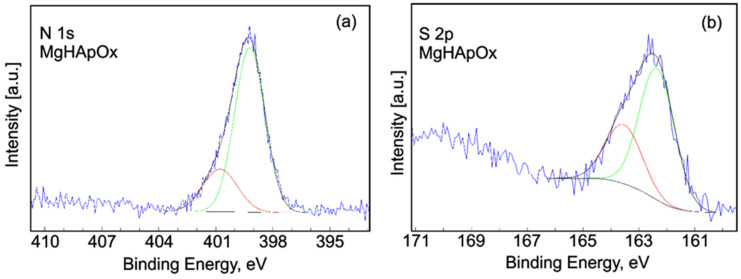
High-resolution XPS spectra and curve-fitting results of N1s (**a**) and S 2p (**b**) for MgHApOx sample.

Figure 15 presents the high-resolution XPS spectra and curve-fitting results of N1s and S 2p for the MgHApOx sample. The high-resolution XPS spectra of N 1s (Figure 15a) showed two distinct signals that were observed at 399.21 and 400.68 eV. The first signal could be assigned to C–N and C–N–C bands. The second signal suggested a protonated N. The high-resolution XPS spectra of S 2p (Figure 15b) showed two distinct signals located at 162.35 and 163.55 eV. The peak of S 2p3/2 for the MgHApOx sample was observed at a binding energy of 162.35 while the peak of S 2p1/2 was observed at a binding energy of 163.55 eV. The doublet separation (2p3/2 − 2p1/2) was equal to 1.2 with a 2:1 area ratio (2p3/2:2p1/2). The two components indicate the presence of C–S bonds [56]. The results of the XPS studies demonstrated that the synthesis method used led to obtaining magnesium-doped hydroxyapatite (MgHAp) and hydroxyapatite doped with magnesium and enriched with amoxicillin (MgHApOx). The results of this study work are in good accordance with the data from the literature [57,58].

**Figure 16:**
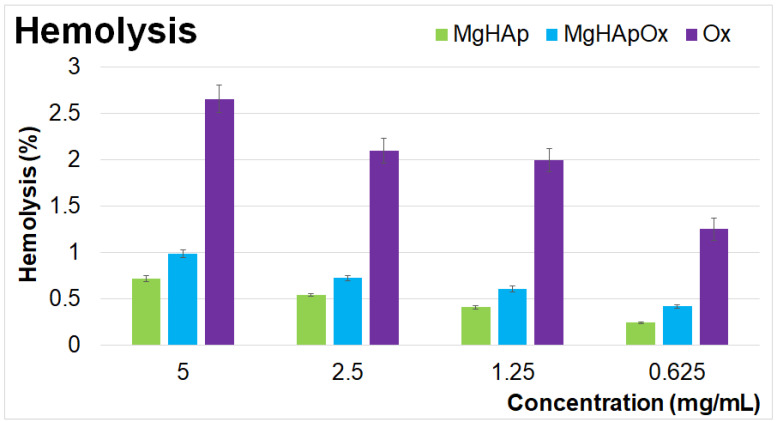
Percentage hemolysis of sheep red blood cells (RBCs) exposed to different concentrations of MgHAp, MgHApOx and Ox suspensions. The statistical analysis of the data was performed using one-way ANOVA. The calculated *p*-values were *p* < 0.002.

The cytotoxicity of MgHAp, MgHApOx and Ox suspensions was evaluated through hemocompatibility and biocompatibility studies. Hemolysis assays are commonly used to assess the potential hemolytic activity of materials. The results of the hemolysis assays provide significant information about the cytotoxic effects of the tested suspensions. The hemolysis index serves as a key parameter in determining the suitability of materials for biomedical applications. This study aims to identify if the tested materials can produce a rupture of the red blood cells (RBCs), which could lead to hemoglobin release and subsequent hemolysis. A low hemolytic index, having a value of less than 5%, suggests that the tested materials are hemocompatible and safe for biomedical use, while a moderate hemolytic index (5–20%) indicates the need for further testing of the materials to ensure safe use. Conversely, a high hemolytic index, having a value higher than 20%, suggests that the materials could pose significant hemolytic risks, making them unsuitable for use in biomedical applications due to their ability to induce potential damage to RBCs and associated negative physiological effects such as inflammation, thrombosis and organ damage. Materials with a low hemolytic index are preferred for use in biomedical applications due to their minimal risk of adverse reactions and high compatibility with other biological systems. In the case of MgHAp and MgHApOx suspensions, the hemocompatibility assays showed that their hemolytic activity was below 1% for both samples, while the hemolytic activity for the Ox suspensions was between 2.5% and 1.25% depending on the tested concentration. Medical drugs and substances are considered non-hemolytic according to national biological safety standards if the hemolysis rate is below 5%. The results of the hemolytic assays of MgHAp, MgHApOx and Ox suspensions are depicted graphically as mean ± SD in Figure 16.

**Figure 17:**
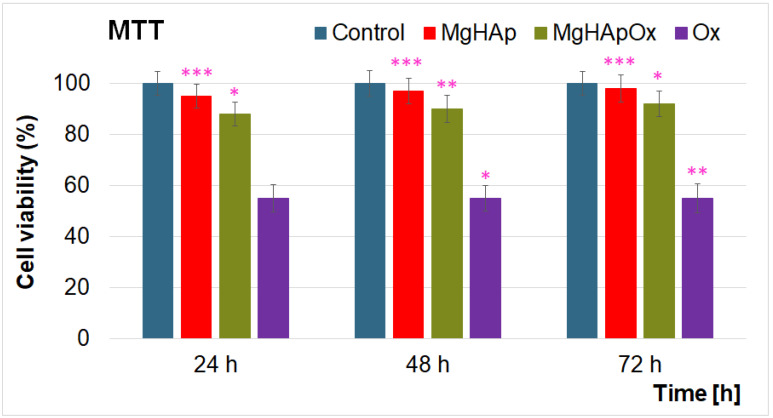
MTT assay of hFOB 1.19 cells incubated with MgHAp, MgHApOx and Ox suspensions for 24, 48 and 72 h. The results are represented as mean ± standard deviation (SD) and are expressed as percentages of control (100% viability). The statistical analysis of the data was performed using one-way ANOVA. The *p*-values indicated are * *p* ≤ 0.002, ** *p* ≤ 0.001, *** *p* ≤ 0.0001.

The hemolytic activity studies revealed that none of the tested concentrations of MgHAp, MgHApOx and Ox induced hemolysis. Additionally, the hemolytic index values obtained for MgHAp and MgHApOx were well within the hemocompatibility acceptable limits for biomaterials. Specifically, MgHAp and MgHApOx suspensions exhibited a hemolytic index with a value below 1%. On the other hand, the results revealed that the hemolysis index of all the samples increased with the sample’s concentration. Notably, MgHAp suspensions demonstrated a lower hemolytic index compared to MgHApOx, likely due to the presence of Ox in the sample. These findings indicate that both MgHAp and MgHApOx nanoparticles have a low hemolytic index, suggesting their potential suitability for being considered for further cytotoxicity assessments in order to confirm their safety for biomedical applications. Further information about the cytotoxicity of MgHAp, MgHApOx and Ox suspensions was attained using the colorimetric MTT assay. For this purpose, the cell viability of hFOB 1.19 cells was assessed following exposure to MgHAp, MgHApOx and Ox suspensions at three distinct time intervals. The results of the MTT assay are presented graphically as mean ± standard deviation (SD) expressed as percentages of control (100% viability) in Figure 17.

The MTT assay results presented in Figure 17 demonstrated that the viability of hFOB 1.19 cells remained above 95% after exposure to MgHAp suspensions for 24, 48 and 72 h. Notably, after 48 and 72 h of exposure, the cell viability increased to 97% and 98%, respectively, when exposed to MgHAp suspensions, revealing their good biocompatibility with hFOB 1.19 cells. These findings align well with previously reported research studies on the biological properties of magnesium-doped hydroxyapatite biocomposites [16,20,59,60,61,62,63,64]. Moreover, these data suggest that MgHAp suspensions are supportive of human osteoblast cell viability and proliferation and could be considered for future biomedical applications [16,20,59,60,61,62,63,64]. Furthermore, the data obtained with the aid of the MTT assay emphasized that the MgHApOx suspensions did not exhibit a cytotoxic effect against hFOB 1.19 cells for any of the tested time intervals. The results depicted a cellular viability lower than in the case of MgHAp suspensions but still above 88%, which is a good rate for a biocompatible material. According to the ISO 10993-1: 2018 standard [65,66,67], biocompatibility is defined as the ability of a material to perform with an appropriate host response in a specific application. Therefore, a viability of over 88% is considered biocompatible due to the fact that it demonstrates a high level of compatibility with living cells, tissues or organisms. The cell viability, in this context, depicts the percentage of cells that remain alive and functional after being exposed to the tested material. A value above 88% indicates that the material does not induce significant cytotoxic effects, meaning it does not damage or induce apoptosis in cells in its vicinity. A viability of over 88% is considered to be a high viability and suggests that the material does not trigger adverse biological responses, such as inflammation, toxicity or immune rejection. These are the most important factors in determining whether a material can safely be integrated and function within a biological system.

On the other hand, the results revealed a slight increase in the cellular viability of hFOB 1.19 cells with an increase in the exposure time, reaching 92% after 72 h. These results showed that the presence of amoxicillin in the MgHAp suspensions did not greatly influence the toxicity of the sample. Furthermore, the results also highlighted that, when exposed to pure Ox suspensions, hFOB 1.19 cells achieved only 55% cell viability. Also, the data suggest that this did not change significantly with the increase of the exposure time. The obtained results agree well with previously reported data on the toxicity of amoxicillin [23,24,25]. Due to its broad-spectrum activity and also relatively good biological properties, amoxicillin has been considered a precursor in the development of novel compounds for biomedical applications.

**Figure 18:**
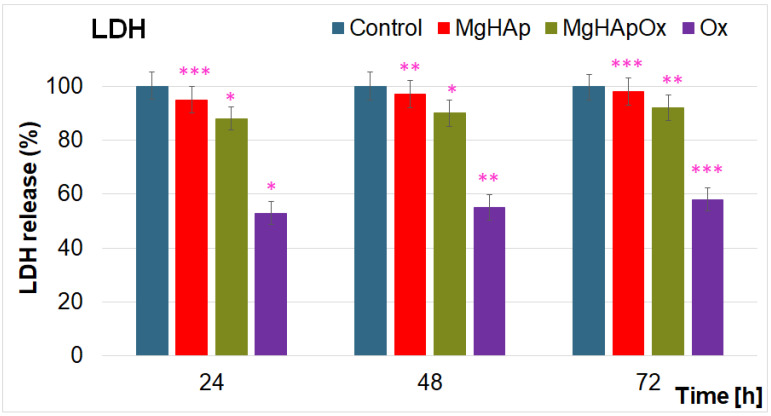
Lactate dehydrogenase (LDH) activity released in the culture medium of hFOB 1.19 cells after the treatment with MgHAp, MgHApOx and Ox suspensions for 24, 48 and 72 h. The results are represented as mean ± standard deviation (SD). The statistical analysis of the data was performed using one-way ANOVA. The *p*-values indicated are * *p* ≤ 0.002, ** *p* ≤ 0.001, *** *p* ≤ 0.0001.

Additional information about the cytotoxic character of the MgHAp, MgHApOx and Ox suspensions was obtained from lactate dehydrogenase (LDH) release studies (Figure 18).

LDH assays determine cell cytotoxicity by detecting LDH release from damaged cells into the culture medium. In this context, a low LDH release index indicates low cytotoxicity and high cell viability, while a high LDH release index suggests significant cell damage. The LDH assay results of MgHAp and MgHApOx suspensions showed no significant difference from the control, indicating that these suspensions did not damage the hFOB 1.19 cell membrane or cause cell necrosis. Additionally, the results of the LDH studies also depicted an LDH value of 53% after 24 h of exposure for the pure Ox suspensions and showed a slight increase at about 58% after 72 h. The findings are consistent with the MTT assay results and demonstrate that both MgHAp and MgHApOx suspensions possess great biocompatibility, making them suitable candidates for the development of novel materials with biomedical applications.

**Figure 19:**
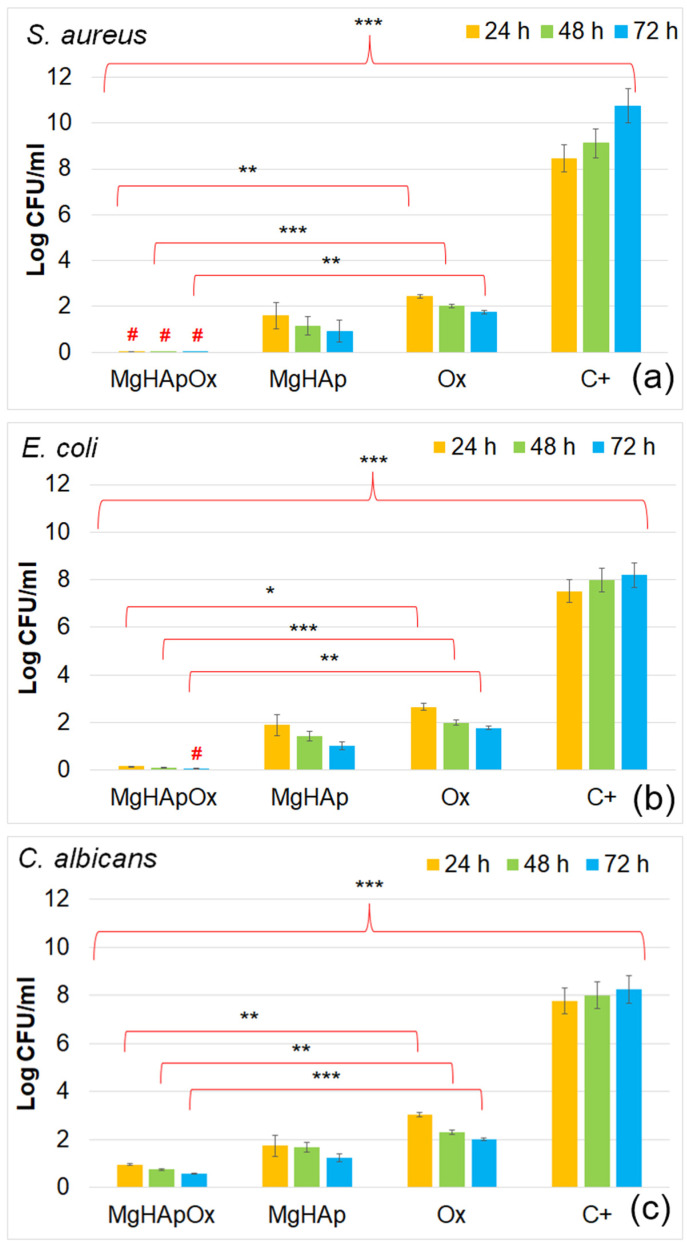
Graphical representation of the logarithmic values of colony forming units (CFU)/mL of *S. aureus* ATCC 25923 (**a**), *E. coli* ATCC 25922 (**b**) and *C. albicans* ATCC 10231 (**c**) microbial strains after 24, 48 and 72 h of exposure to MgHAp, MgHApOx and Ox suspensions. #—depicts the bactericidal properties of the samples. The results are represented as mean ± standard error. Ordinary one-way ANOVA was used for the statistical analysis. The *p*-values indicated are * *p* ≤ 0.002, ** *p* ≤ 0.001, *** *p* ≤ 0.0001.

In recent years, due to the emergence of antibiotic-resistant microbial strains, the development and understanding of the mechanisms and efficacy of novel antimicrobial agents have been an interest of researchers worldwide. Amoxicillin is an antibiotic that has been widely used due to its effectiveness in treating various bacterial infections and has become a crucial part of antimicrobial therapy, particularly against Gram-positive and Gram-negative bacteria. In this context, the development of new antimicrobial agents, such as magnesium-substituted hydroxyapatite (MgHAp), represents a significant advancement in the material science field [9,25,57,68,69,70,71,72,73,74,75,76,77,78,79,80,81,82]. The antimicrobial effects of MgHAp as well as MgHApOx suspensions and pure Ox were evaluated over three different time intervals against one of the most common microbial strains, *Staphylococcus aureus* (*S. aureus*), as well as *Escherichia coli* (*E. coli*) and *Candida albicans* (*C. albicans*). The studies revealed that incorporating amoxicillin into MgHAp had a significant influence in enhancing the antimicrobial effects of MgHAp against *S. aureus*, *E. coli* and *C. albicans*. The results of the in vitro assays are depicted in Figure 19a–c.

The results depicted that the presence of Ox in the MgHAp suspensions conferred a significant increase in the MgHAp antimicrobial activity for all the tested microbial strains. The data showed that the MgHApOx achieved bactericidal properties against *S. aureus* for all the tested time intervals (Figure 19a). In addition, bactericidal activity was depicted for MgHApOx suspensions after 72 h of exposure in the case of *E. coli* (Figure 19b). These results align well with the data reported in the literature that highlights that amoxicillin is less effective against Gram-negative bacteria than Gram-positive ones and has very poor activity against fungal cells [25]. The data also emphasized that the presence of Ox in the MgHApOx samples increased the inhibitory effects of the MgHAp in the case of *E. coli* and *C. albicans*. The results revealed that the CFU values of the tested microbial strains were decreased by the presence of Ox in the case of MgHApOx compared to the values obtained for MgHAp, as well as for the CFU values of the control sample. Magnesium is well known to be a vital element that has an important function in the fundamental nucleic acid chemistry within the cells of all living organisms [68]. While it is widely recognized and studied for its beneficial contribution to bone health and muscle function, recent research studies have also highlighted that it exhibits antimicrobial properties [57,68,69,70,71]. Previously reported studies showed that magnesium-based composites possess important antimicrobial activity against some of the most common pathogens. In this context, magnesium-based composites have been reported to possess antimicrobial properties against *Staphylococcus aureus*, *Pseudomonas aeruginosa*, *Escherichia coli* and *Candida albicans* [57,68,69,70,71]. The antimicrobial effects of these compounds were primarily attributed to the release of magnesium ions, which have the ability to inhibit microbial growth by disrupting bacterial cell membranes [57,68,69,70,71,72]. Additionally, strong antifungal properties were also reported in the case of magnesium-based composites [57,68,69,70,71,72]. Research on the effects of magnesium ions against the fungal pathogen *Candida albicans* has revealed that magnesium ions inhibit fungal cell growth and induce the formation of reactive oxygen species, which exert significant toxic effects on fungal cells [57,68,69,70,71,72,73]. Even though the antimicrobial mechanism of materials is still under investigation, and there is still scarce information in this field, it has been reported that magnesium ions exhibit antimicrobial activity through possible several mechanisms. The primary antimicrobial mechanism reported for magnesium ions is their ability to disrupt the integrity of microbial cell membranes. Another possible mechanism was attributed to magnesium ions’ ability to interfere with the lipid bilayer of microbial cells, leading to increased membrane permeability, which causes cell lysis. On the other hand, it has been reported that magnesium ions can inhibit the replication of microbial DNA by binding to nucleotides or enzymes involved in DNA synthesis, thereby preventing microbial growth. Furthermore, magnesium can act as a cofactor for various enzymes that produce reactive oxygen species (ROS), which can damage microbial cells by oxidizing cellular components like proteins, lipids and DNA. These combined effects contribute to the antimicrobial properties of magnesium ions. On the other hand, amoxicillin exhibits its antimicrobial effects by targeting bacterial cell wall synthesis. Being a beta-lactam antibiotic, amoxicillin has the ability to bind to and inhibit penicillin-binding proteins (PBPs) that are important for the cross-linking of peptidoglycan chains, which are a critical component of bacterial cell walls. This inhibition ability leads to the weakening of the cell wall structure, thus leaving bacteria susceptible to the osmotic pressure, causing cell lysis over time and, eventually, death [9,75,76,77]. Amoxicillin is well known for being particularly effective against a broad spectrum of Gram-positive and some Gram-negative bacteria, making it a widely used antibiotic in the treatment of various bacterial infections [9,75,76,77,78]. The results of the in vitro antimicrobial assays are consistent with previously reported studies on the antimicrobial effects of magnesium-based composites and amoxicillin [9,25,57,68,69,70,71,72,73,74,75,76,77,78,79,80,81,82]. The results demonstrated that the antimicrobial activity of MgHAp and MgHApOx is also dependent on the incubation time and on the type of microbial strain. The data indicated that while the CFU values were low in the early stages of development, their number decreased significantly with exposure time. The superior antimicrobial activity of the MgHApOx suspensions could be attributed both to the release of magnesium ions from the hydroxyapatite matrix as well as to the presence of amoxicillin. The results have suggested that the synergy between the magnesium ions combined with amoxicillin can significantly enhance the antimicrobial efficacy against the tested microbial strains. Magnesium ions are vital in stabilizing bacterial cell walls and membrane structures. When used in combination with amoxicillin, which is a beta-lactam antibiotic that has the ability to disrupt bacterial cell wall synthesis, magnesium ions can help amplify the antibiotic’s effectiveness. This synergistic effect may arise from the ability of magnesium ions to interfere with bacterial defense mechanisms, increasing the cells’ vulnerability to amoxicillin. Additionally, magnesium ions might be able to reduce the bacterial resistance to amoxicillin, leading to treatment that has more effective outcomes in infections. In their studies, Enan et al. [82] also concluded that the AgNPs showed synergistic antimicrobial and anti-biofilm activity when combined with amoxicillin. The results of the biological assays suggested that both MgHAp and MgHApOx suspensions exhibit unique properties. Moreover, the results highlighted that the combination of MgHAp with amoxicillin leads to the development of multifunctional biomaterials ideal for biomedical applications such as bone implants and infection management. The properties that magnesium ions confer to HAp, such as biodegradability, biocompatibility and bone-like mechanical properties, allow the novel materials to have a reduced risk of stress shielding and to promote osteogenesis, making it a suitable candidate for biomedical applications that support bone healing. On the other hand, the broad-spectrum antimicrobial activity of amoxicillin helps prevent the apparition of infections. When integrated into magnesium-doped hydroxyapatite, it can provide localized, continuous drug release, thus reducing the risk of infections and also enhancing the healing process. The synergy between the constituents of magnesium-doped hydroxyapatite enriched with amoxicillin could have the ability to accelerate the process of bone regeneration and prevent infection-related complications. Despite all of these beneficial properties, challenges regarding the optimization of release rates and avoiding antibiotic resistance remain critical for future research studies.

Magnesium plays a crucial role in acid-based processes in the biological environment, contributing significantly to bone calcification and decalcification. Given that half of the body’s magnesium is in bone tissue, where it plays a crucial role in stimulating the growth of new tissue, the composite of magnesium-doped hydroxyapatite and enriched with amoxicillin (MgHApOx) could significantly help regenerate bone tissue after implantation. Moreover, the presence of amoxicillin in the MgHApOx composite can contribute to significantly reducing the risk of postoperative infection. Controlled ionic substitutions can make the hydroxyapatite composition more similar to natural bone, enhancing bioactivity and bone-forming capacity [83]. In contrast, the inclusion of amoxicillin in the composite material added significant value due to its antibacterial, wound healing and anti-inflammatory properties. The research presented in this paper brings new information to the existing research on the controlled substitution of different ions of apatites [84,85]. This study aligns with the recent interest in the use of Mg^2+^ ions as additives in the development of bone biomaterials [84,85,86,87,88]. Moreover, recent studies [16] showed that the presence of chitosan in the magnesium-doped hydroxyapatite/chitosan composite material could have an important contribution due to its antibacterial, cicatrizing and anti-inflammatory properties. The antimicrobial properties of magnesium and magnesium composites have been attributed to different mechanisms, which are similar to those encountered with other metal ions. Previous results [34] on the antimicrobial activity of 10MgHAp suspensions showed that the magnesium-doped hydroxyapatite suspension has a high efficiency in inhibiting biofilm formation for *P. aeruginosa, S. aureus* and *C. albicans* microbial strains, even when used in low concentrations (0.009 mg/mL). Furthermore, the results indicate that the inhibitory effect of MgHAp (x_Mg_ = 0.1) suspensions depends on both incubation time and sample concentration. Studies have shown that corrosion products from modern biocompatible magnesium alloys do not significantly impact human metabolic pathways [89,90]. Additionally, current research [91] has demonstrated that magnesium device implantation causes minimal changes in blood composition without harming excretory organs like the liver or kidneys. Consistent with anterior findings, the colloidal stability of hydroxyapatite suspensions doped with various cations is influenced by different parameters such as hydrodynamic diameters, zeta potential, spectral attenuation and first echo amplitude. Further, the preparation method, the amount of dopant and the surface of the nanoparticles play an important role regarding the stability of hydroxyapatite suspensions doped with different ions [92,93]. At the same time, previous studies [16,33,34,57,58] have shown that the presence of Mg ions in the HAp influences the crystallinity, particle size and the position of the vibration bands associated with phosphate and hydroxyl groups in MgHAp. These characteristics are important for adjusting the properties of nanocomposite materials for use in specific biomedical applications.

The findings from this study suggest that MgHAp and MgHApOsp suspensions could be used in the future development of new antimicrobial agents. The results obtained in this study emphasized that these materials could have significant potential to address the current critical clinical challenges like peri-implantitis and bone defect infections. These novel materials could offer a safe and effective solution in advanced regenerative medicine and implantable medical devices.

## 3. Materials and Methods

### 3.1. Materials

The magnesium-doped hydroxyapatite (MgHAp) and magnesium-doped hydroxyapatite enriched with amoxicillin (MgHApOx) suspension were obtained using the following as reagents: diammonium hydrogen phosphate (NH_4_)_2_HPO_4_, ≥99.0%, Sigma Aldrich, St. Louis, MO, USA), calcium nitrate (Ca(NO_3_)_2_·4H_2_O, ≥99.0%, Sigma Aldrich, St. Louis, MO, USA), magnesium nitrate hexahydrate (Mg(NO_3_)_2_·6H_2_O, 99.97%; Alpha Aesar, Kandel, Germany) and amoxicillin (C_16_H_19_N_3_O_5_S, 95.0–102.0%, Sigma Aldrich, St. Louis, MO, USA).

### 3.2. Synthesis of Magnesium-Doped Hydroxyapatite Enriched with Amoxicillin Suspensions

Both suspensions (MgHAp and MgHApOx) were developed using an adapted co-precipitation method, in agreement with our previous work [34,94]. For both syntheses, the [Ca + Mg]/P ratio was set at 1.67, and the magnesium concentration was x_Mg_ = 0.1 [34,94]. In the following, we will only describe the synthesis method of MgHApOx because this is similar to that of MgHAp. Therefore, the solution containing amoxicillin, magnesium and calcium was obtained by dissolving the specific precursors in a beaker. Then, the obtained solution was added drop by drop in the phosphate solution. Then, the mixture was well stirred (250 rpm) for 12 h at 80 °C on a magnetic stirrer [34,94]. Finally, the obtained mixture was centrifuged using a Hettich Universal 320 centrifuge (Hettich, Tuttlingen, Germany; operated at 6000 rpm for 12 min/cycle) and dispersed in deionized water (4 times). Prior to X-ray diffraction (XRD), energy-dispersive X-ray spectroscopy (EDS), X-ray photoelectron spectroscopy (XPS) and scanning electron microscopy (SEM) analyses, both suspensions were vacuum-dried, and the resulting powders (MgHAp and MgHApOx) were subjected to further analysis.

### 3.3. Phisycochemical Characterization of Samples

#### 3.3.1. Ultrasound Studies

**Figure 20:**
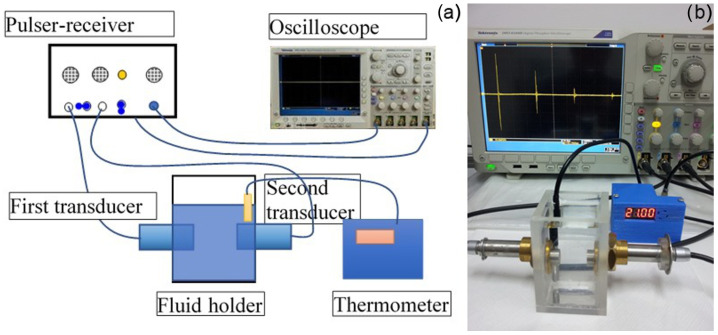
The schematic representation (**a**) and the image (**b**) of US experimental setup [95].

In order to evaluate the stability of the concentrated suspensions of MgHAp and MgHApOx, ultrasound studies were carried out. For this purpose, 100 mL of MgHAp and MgHApOX suspension were stirred continuously at room temperature for 15 min at 700 rpm in order to obtain a good homogeneity of the solid particles. After continuous stirring, the 100 mL were transferred to a container special transparent cubic. Two ultrasonic coaxial transducers were immersed in the suspension and distanced 16 mm face-to-face. The axis of the transducers was 29 mm from the flat bottom of the container box. Immediately after stopping the magnetic stirrer, the acquisition of ultrasonic signals began. In the case of the MgHAp suspension, 1000 ultrasonic signals were acquired, while for the MgHApOx suspension, 900 ultrasonic signals were acquired, recorded every 5 s from the oscilloscope. Each recorded signal is an average of 32 signals on the oscilloscope, reducing experimental noise. For this study, the US measurements were performed using two identical ultrasonic transducers with a central frequency of 5 MHz (GE’s Krautkramer, GE, Boston, MA, USA). One transducer generated and received ultrasonic signals in the fluid, while the second acted solely as a receiver. The signals were amplified and processed with a pulse receiver and a Tektronix DPO 4014B oscilloscope (Tektronix, Inc., Beaverton, OR, USA), while a digital thermometer measured the fluid temperature. All the experimental US data were recorded and analyzed using proprietary software [95,96,97]. For better accuracy, both the schematics and image of the US experimental setup are revealed in Figure 20 [95].

#### 3.3.2. Zeta (ζ)-Potential Measurements

Zeta (ζ)-potential studies were performed with the aid of an SZ-100 Nanoparticle Analyzer (Horiba-SAS France, Longjumeau, France) instrument at room temperature. To ensure the accurate characterization of the particle surface charge, prior to measurement, the suspensions were systematically diluted tenfold in deionized water in order to minimize the particle concentration effects on ζ-potential values [32]. For reproducibility and reliability, three independent measurements were performed for each sample.

#### 3.3.3. X-ray Diffraction

X-ray diffraction (XRD) of MgHAp and MgHApOx were investigated using a Bruker D8 Advance diffractometer with CuKα radiation (λ = 1.5418 Å) (Bruker, Karlsruhe, Germany) equipped with a LynxEye™ 1D high-efficiency one-dimensional linear detector. Data were acquired in the 2θ range of 10–60° with a step size of 0.02° and a time of 5 s per step. JCPDS # 09–432 and JCPDS # 39-1832 were used to confirm the various peaks developed in the apatite and amoxicillin. The average crystallite size (D) of the samples was calculated using Scherrer’s formula [98]:

D = Kλ/βcosθ(1)

where K is Scherrer constant, λ is wavelength of the X-rays, β is the full width at half maximum (FWHM) and θ is the diffraction angle of the XRD spectra.

The hexagonal crystal parameters were calculated from the equation of Bragg’s reflection [99]. The unit cell volume (Å^3^) was also estimated [100].

#### 3.3.4. Scanning Electron Microscopy

The morphology features of the samples (MgHAp and MgHApOx) were investigated by scanning electron microscopy (SEM) with HITACHI S4500 equipment (Hitachi, Tokyo, Japan) in order to evaluate the influence of amoxicillin on the MgHAp morphology. The parameter values used for the obtaining of SEM images were ETD detector, spot of 3.5 and an accelerating voltage of 20 kV. Furthermore, the mean particle size was obtained after measuring about 500 nanoparticles. Also, information regarding chemical composition of MgHAp and MgHApOx was obtained by performing energy-dispersive X-ray spectroscopy (EDS) studies. For this purpose, an EDS system was attached to the SEM equipment.

#### 3.3.5. Fourier Transform Infrared Spectroscopy

The Fourier transform infrared (FTIR) spectroscopy in attenuated total reflectance (ATR) mode was employed in order to evaluate the vibrations of functional groups present in MgHAp, MgHApOx and Ox. The FTIR studies were performed with a Perkin Elmer spectrometer equipped with a Universal Diamond/KRS-5 (Waltham, MA, USA). All the FTIR spectra were collected in the 450–2500 cm^−1^ spectral range under ambient conditions. The parameters used for the FTIR measurements were: 32 scans with a resolution of 4 cm^−1^. The second derivative spectra and the deconvoluted spectra of the MgHAp, MgHApOx and amoxicillin were achieved for the 900–1200 cm^−1^ spectral domain [38].

#### 3.3.6. X-ray Photoelectron Spectroscopy

For this study, the XPS data were collected using a SCIENCE SES 2002 system (Scienta Omicron, Taunusstein, Germany) equipped with a monochromatic Al Kα X-ray source (hν = 1486.6 eV). The binding energy for the sp2 carbon (C 1s) peak observed at 284.8 eV was used as reference. The XPS data analysis was performed using Casa XPS version 2.3.14 software.

### 3.4. In Vitro Biological Assays

#### 3.4.1. Hemolysis Assay

The biological properties of MgHAp, MgHApOx and Ox suspensions were assessed by hemolysis assay using sheep red blood cells (RBCs). The experiments were done according to the previously reported methodology by Miyaji et al. [101] with the modifications described in [95]. For this purpose, various sample concentrations diluted in a 0.9% NaCl solution were mixed with an equal volume of erythrocyte suspension, incubated at a temperature of 37 °C for 30 min and then centrifuged. Triton X-100 and PBS were used as positive and negative controls, respectively. The hemolytic index (HI%) was quantified from the absorbance of the supernatant measured at 540 nm with a FlexStation 3 UV-VIS spectrophotometer (Molecular Devices, San Jose, CA, USA) with the following equation:

(2) $$\mathrm{Hemolysis}\;(\%)=\frac{V_{ODsample}-V_{ODnegativecontrol}}{V_{ODpositivecontrol}-V_{ODnegativecontrol}}\times 100$$

#### 3.4.2. Colorimetric Test Assay 3-(4,5-Dimethylthiazol-2-yl)-2,5-diphenyltetrazolium Bromide (MTT) Assay

The cytotoxicity of MgHAp, MgHApOx and Ox suspensions was determined using human fetal osteoblastic cells (hFOB 1.19) according to the method reported by Ciobanu et al. [95]. For this purpose, the cells were cultured using Dulbecco Modified Eagle’s Medium. The culture was grown in a humidified atmosphere with 5% CO_2_ at a temperature of 37 °C and seeded at a density of 3 × 10^4^ cells/cm^2^. The cell viability was evaluated after 24, 48 and 72 h exposure intervals with the aid of the colorimetric test assay 3-(4,5-dimethylthiazol-2-yl)-2,5-diphenyltetrazolium bromide (MTT; Sigma-Aldrich, USA). Following each exposure period, the culture medium was removed, and the cells were incubated with 1 mg/mL MTT for 4 h at 37 °C. The absorbance values measured at 595 nm using a FlexStation 3 microplate reader were used to quantify the cell viability. The values of the cell viability were calculated relative to the control sample (set at 100%) and presented graphically as mean ± SD.

#### 3.4.3. Lactate Dehydrogenase (LDH) Release Measurement

The cytotoxicity of the MgHAp, MgHApOx and Ox suspensions was also assessed through a Lactate dehydrogenase (LDH) release measurement with the aid of Cytotoxicity Detection KitPLUS (Roche, Atlanta, GA, USA). The experiments were performed according to the manufacturer’s instructions. After the MgHAp, MgHApOx and Ox suspensions’ incubation with the HeLa cells, 50 µL of the culture supernatant was mixed with 50 µL of reaction mixture containing the catalyst and dye solution and incubated in the dark for 30 min. The LDH was determined from the absorbance values measured at 485 nm using a Tecan GENios instrument.

#### 3.4.4. In Vitro Antimicrobial Assay

The antimicrobial properties of MgHAp, MgHApOx and Ox were evaluated in vitro using the following common reference strains: *Staphylococcus aureus* ATCC 25923, *Escherichia coli* ATCC 25922, and *Candida albicans* ATCC 10231 (all obtained from ATCC, Old Town Manassas, VA, USA). The antimicrobial testing was conducted as described in [102], with microbial cultures standardized to 0.5 McFarland. The MgHAp, MgHApOx and Ox samples were inoculated with 1.5 mL of microbial suspensions at a concentration of 5 × 10^6^ CFU/mL, prepared in phosphate-buffered saline (PBS) and incubated for 24, 48 and 72 h. The positive control was chosen as the free microbial cultures (C+). Suspensions were collected at 24, 48 and 72 h, plated on LB agar medium and incubated for 24 h at 37 °C to determine the CFU/mL count. All experiments were conducted in triplicate, and the data were presented graphically as mean ± SD. The statistical analysis was performed using a single-factor ANOVA test.

#### 3.4.5. Statistical Analysis

All the data from the biological assays were presented as mean ± SD. Unless stated otherwise, the graphs show the average values with error bars representing the standard deviation from at least three replicates per material. Statistical analysis was conducted using ANOVA, with significant differences between groups defined by a *p*-value of *p* < 0.05.

## 4. Conclusions

In this study, magnesium-doped hydroxyapatite (MgHAp) and magnesium-doped hydroxyapatite enriched with amoxicillin (MgHApOx) have been successfully suspensions synthesized for the first time by an adapted co-precipitation method. The aim of this study was focused on the complex characterization of MgHAp and MgHApOx suspensions from both physico-chemical and biological point of view. The influence of amoxicillin on the stability, structural, morphological and biological features of MgHAp sample was evaluated. The obtained suspensions were analyzed by ultrasound measurements, XRD, XPS, SEM, FTIR and EDS studies. Their in vitro biological features were also evaluated by hemolysis assay, MTT assay, lactate dehydrogenase (LDH) release measurement and antimicrobial assay. The XRD analysis confirms the replacement of Ca^2+^ ions with Mg^2+^ ions in the HAp lattice and a significant incorporation of amoxicillin into the MgHAp lattice. XRD and SEM investigations revealed crystalline nanoparticles. The average crystallite size determined from XRD examination increased from 15.31 nm for MgHAp to 17.79 nm for the MgHApOx sample. The crystallite sizes obtained from SEM were consistent with the XRD results. EDS and XPS certified the presence of Mg in the HAp lattice as well as the presence of amoxicillin constituents in the MgHAp lattice. Furthermore, the FTIR results suggest the presence of functional phosphate groups of HAp in both samples. Moreover, the FTIR results prove the presence of amoxicillin in the MgHApOx. Biological studies revealed that MgHAp and MgHApOx nanoparticles exhibit good biocompatibility towards hFOB 1.19 cells and also antimicrobial properties against *S. aureus, E. coli* and *C. albicans*. The hemolysis experiments indicated a hemolytic index of less than 1% for both MgHAp and MgHApOx nanoparticles, and a hemolytic index above 2.5% confirming their suitability for biomedical applications. On the other hand, cell viability studies showed that hFOB 1.19 cells maintained a viability of over 95% in the case of MgHAp nanoparticles above 88% in the case of the MgHApOx nanoparticles with increased viability observed over the extended incubation periods, while the cell viability of Ox powders was around 55%. Additionally, the LDH studies confirmed that exposure to MgHAp and MgHApOx nanoparticles did not compromise the integrity of the hFOB 1.19 cell membranes. These findings emphasize the potential of MgHAp and MgHApOx nanoparticles in the development of future materials, with applications in the biomedical area. Future research on magnesium-doped hydroxyapatite enriched with amoxicillin should be focused on drug loading, targeted delivery and overcoming antimicrobial resistance while ensuring biocompatibility (in vitro and in vivo) and long-term stability.

## Figures and Tables

**Table 1:** XRD parameters of MgHAp and MgHApOx samples.

Sample	Average Crystal Size (nm)	d-Spacing (nm)	Lattice Parameters (Å)		Unit Cell Volume (Å^3^)
			c-Axis	a-Axis	
MgHAp	15.31	3.40	6.85	9.39	605
MgHApOx	17.79	3.43	6.87	9.41	608

## Data Availability

The original contributions presented in the study are included in the article, further inquiries can be directed to the corresponding authors.
